# Low perioperative hemoglobin as an independent detrimental predictor of functional outcome after basal ganglia hemorrhage: development and validation of a composite prognostic model

**DOI:** 10.3389/fnhum.2025.1745109

**Published:** 2025-12-30

**Authors:** Xia Li, Lijun Zhang, Zhikun Yang, Jian Ding, Yanfei Yu, Shuo Yang, Feng Duan, Yi Dong

**Affiliations:** 1 Department of Critical Care Medicine, The Affiliated Hospital of Qingdao University, Qingdao, China; 2 Department of Neurosurgery, Qingdao Central Hospital, University of Health and Rehabilitation Sciences, Qingdao, China; 3 Department of Radiology, The Affiliated Hospital of Qingdao University, Qingdao, China; 4 Department of Neurology, The Affiliated Hospital of Qingdao University, Qingdao, China

**Keywords:** basal ganglia hemorrhage, Hemoglobin, perioperative management, predictive model, prognosis

## Abstract

**Background:**

Basal ganglia hemorrhage (BGH) is a life-threatening neurosurgical emergency associated with substantial mortality and disability. Accurate postoperative prognosis assessment remains challenging due to multifactorial influences. Hemoglobin (HB), as the key determinant of oxygen delivery, may play a critical role in neurological recovery, yet the prognostic significance of perioperative HB fluctuations in BGH has not been fully elucidated.

**Methods:**

A retrospective cohort of 213 surgically treated BGH patients from 2020 to 2023 was analyzed. Perioperative HB indices, including preoperative (Pre-HB), postoperative (Post-HB), and mean HB (Mean-HB) levels, were evaluated alongside clinical data. Functional outcome at 6 months was determined based on the modified Rankin Scale (mRS). Least absolute shrinkage and selection operator (LASSO) regression together with multivariate logistic regression models were utilized to screen for independent risk variables, followed by construction of a composite predictive model. Model discrimination, calibration, and evaluation of the model’s clinical applicability were conducted using receiver operating characteristic (ROC) analysis, calibration plots, and decision curve analysis (DCA).

**Results:**

Patients with poor prognosis exhibited significantly lower Pre-HB, Post-HB, and Mean-HB levels (all *P* < 0.05). Multivariate analysis confirmed these variables as independent predictors of adverse outcome. The proposed model provides a practical and data-driven tool that demonstrated good predictive performance (AUC = 0.84) in a single-center retrospective cohort. Calibration and DCA demonstrated good consistency and potential clinical applicability.

**Conclusion:**

Perioperative declines in HB are independently associated with poor postoperative outcomes in BGH. The proposed HB-integrated model provides a reliable, dynamic tool for individualized risk prediction, facilitating precision perioperative management and optimized recovery strategies.

## 1 Introduction

Basal ganglia hemorrhage (BGH) is defined as bleeding within the basal ganglia region of the brain, typically resulting from hypertension and other contributing factors (Potts and Riina, 2014). BGH represents one of the most common neurosurgical emergencies encountered in clinical practice (Svensson et al., 2020). Despite recent advancements in the treatment of BGH, its high mortality and disability rates continue to pose significant global health challenges. Surgical intervention has been reported as an effective approach that may improve patient survival outcomes (Hazra et al., 2023; Lu et al., 2018; Zhang et al., 2023). Nevertheless, predicting postoperative prognosis remains a complex issue in clinical management. Various factors, such as the volume of bleeding, hematoma location, underlying patient comorbidities, and postoperative complications, may influence recovery and quality of life.

Hemoglobin serves as the primary oxygen transport protein in red blood cells, and fluctuations in its levels directly influence the body’s oxygen supply capacity (Ahmed et al., 2020). In patients with BGH, a low HB level often interacts with various factors such as systemic responses to hemorrhage, hypoxia, and inflammatory reactions, which can significantly affect patient prognosis (Lu et al., 2022; Wei et al., 2019). Particularly during postoperative recovery, anemia may exacerbate the patient’s weakened condition, impair immune responses, compromise organ function, and increase the incidence of postoperative complications (Fonseca et al., 2021; Roh et al., 2024). Consequently, early detection and intervention for low HB levels may enhance the postoperative outcomes of patients with BGH. Recent evidence suggests that HB levels are associated with outcomes in various cerebrovascular diseases. For instance, lower HB levels have been linked with postoperative cerebral infarction in moyamoya disease (Wu et al., 2024), poor prognosis after intracerebral hemorrhage (Acosta et al., 2021), and worse functional outcomes in deep spontaneous hemorrhage, including basal ganglia involvement (Wu et al., 2024; Zhang et al., 2022; Zheng et al., 2019). These studies highlight the potential role of HB as a modifiable prognostic biomarker in neurosurgical settings. Specifically, low HB levels may exacerbate disease progression, delay recovery, and even increase the risk of mortality (Park et al., 2016; Wang et al., 2019).

Although studies have investigated the relationship between HB levels and the prognosis of patients with other cerebrovascular diseases, the specific role of low HB in patients with BGH has not been adequately addressed or systematically summarized. Most existing predictive models primarily focus on clinical factors such as bleeding volume and Glasgow Coma Scale (GCS) scores, lacking a comprehensive integration of HB levels. Therefore, it is crucial to develop a postoperative prognostic prediction model that incorporates low HB levels. By integrating HB levels with other pertinent clinical variables, we can construct a more precise prognostic assessment tool, which will facilitate clinical decision-making and enhance treatment optimization.

The objective of this study is to examine the correlation between low HB levels and the postoperative prognosis of patients with BGH, as well as to construct a predictive model based on HB levels and other clinical indicators for assessing postoperative outcomes in these patients. A retrospective analysis of clinical data from patients with basal BGH will be conducted to evaluate the role of low HB as a predictor of postoperative survival, recovery, and complications. Furthermore, this study will identify key variables strongly associated with postoperative prognosis through multivariate analysis and incorporate them into the development of a comprehensive predictive model. Through this research, we aim to provide a novel prognostic assessment tool for the clinical management of patients with BGH, enabling physicians to more accurately assess patient risk preoperatively and develop personalized treatment plans to optimize postoperative recovery.

## 2 Materials and methods

### 2.1 Patient enrollment

We conducted a retrospective analysis of 213 patients with BGH admitted to the Department of Neurosurgery at the Affiliated Hospital of Qingdao University between 2020 and 2023. The inclusion criteria were as follows: (1) imaging examination results consistent with the diagnostic criteria for BGH; (2) age ≥ 20 years; (3) hematoma volume < 30 mL; (4) patients who underwent surgical intervention; (5) availability of complete perioperative clinical and laboratory data, including preoperative and postoperative hemoglobin measurements; (6) no use of medications known to influence hemoglobin concentration or erythropoiesis (e.g., iron supplements, erythropoietin, corticosteroids, or cytotoxic agents) within the preceding 3 months. The exclusion criteria were as follows: (A) admission more than 24 h after symptom onset; (B) history of chronic inflammatory conditions, hematological disorders (excluding iron-deficiency anemia), or autoimmune diseases; (C) use of anticoagulants or immunosuppressive agents; (D) infection within the past 2 weeks; (E) history of stroke, traumatic brain injury, or craniotomy; (F) history of hematological disorders or malignancy. This study was approved by the Institutional Ethics Committee (Approval No. QYFY WZLL 29979). All participants in this retrospective study were anonymized, and the authors did not have access to any information that could identify individual participants during or after data collection.

### 2.2 Data collection

Demographic and clinical variables were collected from the institutional electronic medical record system, including gender, age, smoking history, alcohol history, BMI, systolic blood pressure (SBP), diastolic blood pressure (DBP), hypertension, hyperlipidemia, diabetes, GCS score at admission. Additionally, HB levels were recorded at various perioperative time points, including Pre-HB, Post-HB, minimum hemoglobin during the perioperative period (Min-HB), maximum hemoglobin (Max-HB), and Mean-HB. Pre-HB was defined as the first HB measurement obtained upon hospital admission without any surgical or transfusion intervention. Furthermore, data from all 213 patients were analyzed to evaluate disease progression and functional outcomes using the modified Rankin Scale (mRS) 6 months post-treatment. Based on their mRS scores, patients were categorized into two groups: the Good Prognosis Group (mRS < 3, *n* = 160) and the Adverse Prognosis Group (mRS ≥ 3, *n* = 53). Missing data for continuous variables (<5% of total data) were handled using multiple imputation based on chained equations (MICE) to minimize bias. Sensitivity analyses were performed to ensure the robustness of results, confirming that imputation did not materially affect statistical significance or model performance.

### 2.3 The LASSO model

Least absolute shrinkage and selection operator (LASSO) regression was employed to select the most informative predictor variables for constructing the model, particularly suited for scenarios with small sample sizes and high-dimensional data. The R packages “glmnet,” “corrplot” and “caret” were utilized, along with five-fold cross-validation, to determine the optimal λ value and achieve robust variable selection results.

### 2.4 Statistical analysis

All statistical analyses were performed using SPSS software (version 26.0) and R software (version 4.4.3). Continuous data following a normal distribution were described as the mean ± standard deviation (SD), whereas non-normally distributed data were expressed as the median and interquartile range (IQR, 25th–75th percentiles). Categorical variables were reported as counts and percentages. Group comparisons were executed through the Student’s *t*-test for normally distributed data, while the Kruskal–Wallis test was employed for non-normally distributed variables, and the Chi-square test was applied for qualitative variables. Multivariate logistic regression was utilized to explore independent prognostic indicators related to poor outcomes. ROC analysis was conducted to assess the discriminative capacity of the predictive model, and the AUC was determined. Model calibration was examined using calibration curves and DCA to evaluate its clinical utility. A two-tailed *P*-value below 0.05 was considered to indicate statistical significance.

## 3 Results

### 3.1 Baseline characteristics and HB level distribution

According to the inclusion and exclusion criteria, 213 patients with BGH were enrolled in this study, with 53 patients (24.88%) in the Adverse Prognosis Group and 160 patients (75.12%) in the Good Prognosis Group. The baseline characteristics of the patients included a mean age of 51.94 ± 9.49 years, with 77 males (36.15%) and 136 females (63.85%). Among the patients, hematoma occurred unilaterally in 174 cases (81.69%) and bilaterally in 39 cases (18.31%). Based on the Glasgow Coma Scale (GCS) scores, 117 patients (54.93%) had scores ranging from 3 to 8, 64 patients (30.05%) had scores from 9 to 12, and 32 patients (15.02%) had scores from 13 to 15.

Comparison of baseline characteristics between the Adverse Prognosis Group and the Good Prognosis Group showed no significant differences in clinical features such as gender, age, smoking history, alcohol history, BMI, SBP, DBP, hypertension, hyperlipidemia, diabetes and hemorrhage side (*P* > 0.05), indicating that these factors had no significant impact on prognosis. However, the GCS scores in the Adverse Prognosis Group were significantly lower than those in the Good Prognosis Group (*P* < 0.001), suggesting a significantly poorer level of consciousness among patients with unfavorable outcomes. Furthermore, Pre-HB levels (126.81 ± 16.95 g/L vs. 132.81 ± 18.26 g/L), Post-HB (127.04 ± 17.45 g/L vs. 133.64 ± 17.61 g/L), Min-HB (114.74 ± 13.54 g/L vs. 119.85 ± 14.73 g/L), Max-HB (140.01 ± 15.54 g/L vs. 147.17 ± 14.07 g/L), and Mean-HB during hospitalization (127.79 ± 12.95 g/L vs. 132.77 ± 12.07 g/L) were significantly lower in the Adverse Prognosis Group compared with the Good Prognosis Group (all *P* < 0.001), suggesting that reduced perioperative hemoglobin levels are closely associated with unfavorable outcomes (Table 1).

**TABLE 1:** Comparison between the clinical data of patients with basal ganglia hemorrhage.

Characteristics	The Adverse Prognosis Group (mRS ≥ 3; *n* = 53)	The Good Prognosis Group (mRS < 3; *n* = 160)	*P*-value
Gender			0.054
Male, *n* (%)	25 (47.2%)	52 (32.5%)	
Female, *n* (%)	28 (52.8%)	108 (67.5%)	
Age	52.66 ± 9.1881	49.719 ± 9.8525	0.057
Smoking history, *n* (%)	22 (41.5%)	44 (27.5%)	0.056
Alcohol history, *n* (%)	15 (28.3%)	33 (20.6%)	0.246
BMI (kg/m^2^)	23.023 ± 1.9692	22.55 ± 2.0324	0.141
SBP (mmHg)	125.79 ± 18.349	125.94 ± 20.572	0.962
DBP (mmHg)	77.566 ± 10.85	78.325 ± 12.964	0.701
Hypertension, *n* (%)	38 (71.7%)	92 (57.5%)	0.066
Hyperlipemia, *n* (%)	35 (66%)	95 (59.4%)	0.389
Diabetes, *n* (%)	7 (13.2%)	29 (18.1%)	0.408
Pre-HB (g/L)	126.81 ± 16.95	132.81 ± 18.26	0.029^†^
Post-HB (g/L)	127.04 ± 17.45	133.64 ± 17.61	0.018^†^
Min-HB (g/L)	114.74 ± 13.54	119.85 ± 14.73	0.021^†^
Max-HB (g/L)	140.01 ± 15.54	147.17 ± 14.07	0.003^†^
Mean-HB (g/L)	127.79 ± 12.95	132.77 ± 12.07	0.014^†^
Hemorrhage side			0.078
1	39 (73.6%)	135 (84.4%)	
2	14 (26.4%)	25 (15.6%)	
GCS score at admission			<0.001^†^
3–8	34 (64.2%)	83 (51.88%)	
9–12	18 (34.0%)	46 (28.75%)	
13–15	1 (1.8%)	31 (19.37%)	

† Indicates statistical significance. Hemorrhage side was coded as “1 = unilateral” and “2 = bilateral.” This coding has now been clarified in the Section “2 Materials and methods” and noted in the footnote of Table 1.

### 3.2 LASSO regression variable selection and risk model construction

**FIGURE 1:**
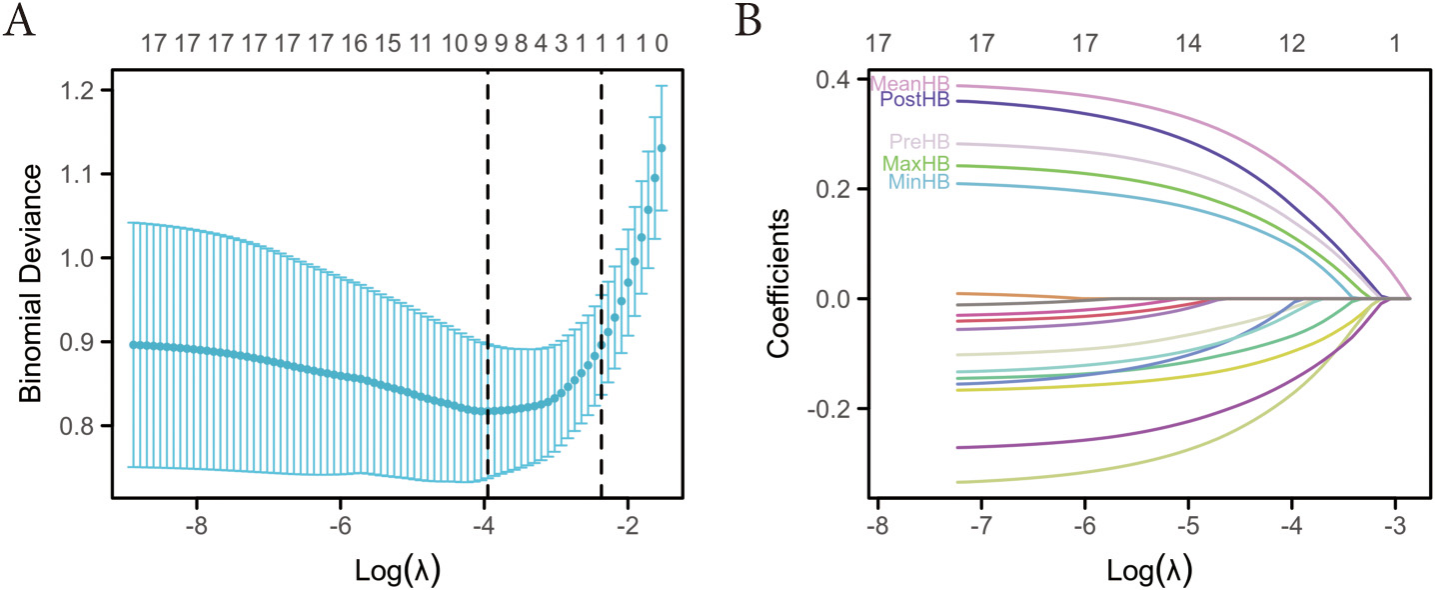
Variable selection using LASSO regression in BGH patients. **(A)** Cross-validation curve showing binomial deviance versus log(λ). The optimal λ was determined by the minimum deviance and 1-SE criteria; **(B)** LASSO coefficient profiles of perioperative hemoglobin variables. Pre-HB, Post-HB, Min-HB, Max-HB, and Mean-HB were retained as key predictors for model construction.

To identify key indicators associated with poor functional outcome, the LASSO regression method was applied to screen 17 candidate variables. Figure 1A demonstrates the trend of model deviation as the penalty coefficient (λ) changes. As the λ value increased, the coefficients of some variables gradually approached zero, and the model deviation first decreased and then increased. Ultimately, based on the minimum deviation criterion and the 1 standard error rule, five key HB-related variables were selected: Pre-HB, Post-HB, Min-HB, Max-HB, and Mean-HB (Figure 1B). Among the five HB variables initially identified by LASSO, Min-HB and Max-HB were excluded from the final model owing to their nonsignificant contribution (*P* > 0.05) and high perioperative variability, while Pre-HB, Post-HB, and Mean-HB remained as independent predictors.

**FIGURE 2:**
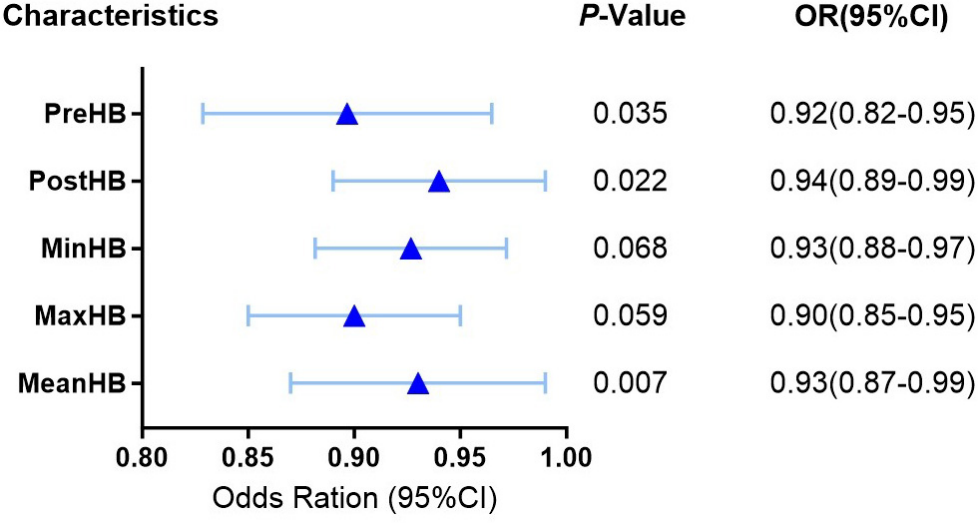
Multivariate logistic regression of perioperative hemoglobin indices. Forest plot showing the association between hemoglobin indices and poor functional outcomes (mRS ≥ 3). Pre-HB, Post-HB, and Mean-HB were independently associated with reduced risk of poor prognosis (*P* < 0.05).

In the multivariate logistic regression analysis, all HB indices demonstrated an inverse relationship with the risk of poor functional outcome, meaning that as HB levels increased, the likelihood of poor prognosis decreased. Specifically, Pre-HB (*P* = 0.035, OR = 0.92, 95% CI: 0.82–0.95), Post-HB (*P* = 0.022, OR = 0.94, 95% CI: 0.89–0.99), and Mean-HB (*P* = 0.007, OR = 0.93, 95% CI: 0.87–0.99) were significantly associated with a reduced risk of poor functional outcome. Min-HB (*P* = 0.068, OR = 0.93, 95% CI: 0.88–0.97) and Max-HB (*P* = 0.059, OR = 0.90, 95% CI: 0.85–0.95) approached statistical significance, indicating that fluctuations in HB levels may play a role in changing patient outcomes (Figure 2).

### 3.3 Construction and validation of the risk prediction model

**FIGURE 3:**
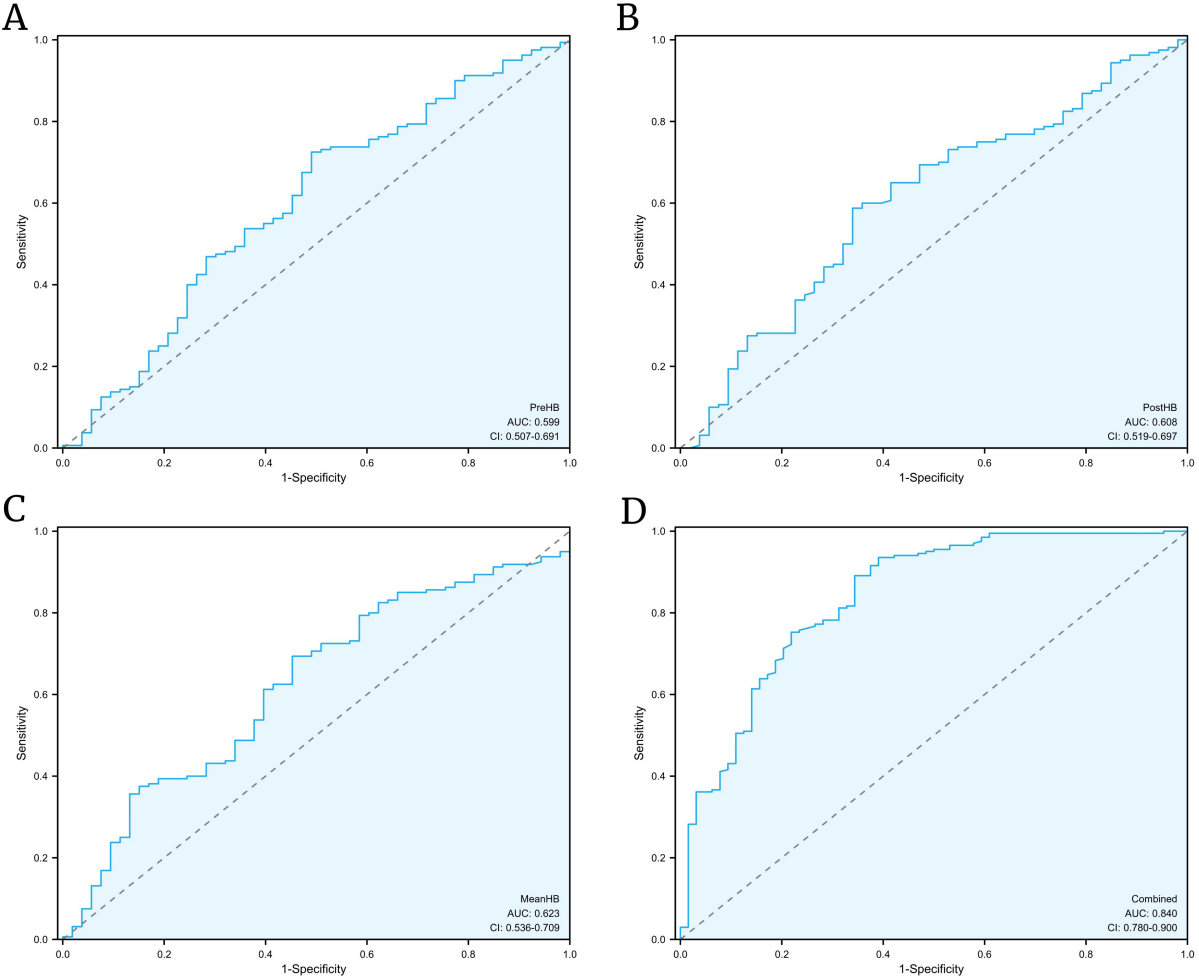
Receiver operating characteristic (ROC) analysis of hemoglobin indices and the composite predictive model. **(A–C)** ROC curves for single indicators–Pre-HB, Post-HB, and Mean-HB–showing moderate predictive performance (AUC = 0.599–0.623); **(D)** ROC curve for the composite model combining HB indices with clinical factors, demonstrating superior discrimination (AUC = 0.840).

Based on the key HB indicators selected by LASSO regression and logistic regression (Pre-HB, Post-HB, Mean-HB), a risk prediction model was developed. ROC curve analysis (Figures 3A–C) showed that the AUC of single HB indices (Pre-HB, Post-HB, Mean-HB) was 0.599 (95% CI: 0.507–0.691), 0.604 (95% CI: 0.517–0.690), and 0.623 (95% CI: 0.536–0.709), respectively, indicating moderate discrimination. By combining these three HB indices with clinical characteristics (such as the side of hematoma occurrence and GCS scores), the “composite model” significantly improved the AUC to 0.840 (95% CI: 0.780–0.900) (Figure 3D), showing that the model outperformed single indices in terms of sensitivity and specificity.

**FIGURE 4:**
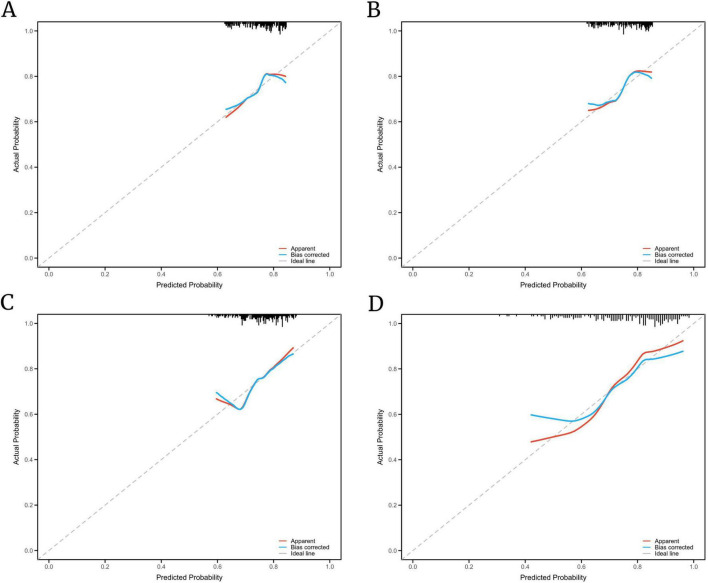
Calibration performance of the predictive models. **(A–C)** Calibration curves for single-indicator models showing mild deviation from the ideal 45° line; **(D)** the composite model shows optimal alignment between predicted and observed outcomes, indicating superior calibration.

To further evaluate the calibration performance of the prediction models, calibration curves were generated for each model. Figure 4 demonstrate the apparent and bias-corrected calibration plots after 1,000 bootstrap resamples. Overall, the predicted probabilities were in good agreement with the observed outcomes across most risk ranges, indicating acceptable calibration. As illustrated in Figures 4A–C, the single-indicator models demonstrated slight deviations from the ideal 45° reference line, particularly in the higher predicted probability range, reflecting a mild tendency to overestimate the risk of poor outcome. In contrast, the combined model showed the closest agreement between predicted and observed probabilities across most probability intervals, especially within the moderate- to high-risk range, indicating superior calibration stability and overall predictive consistency (Figure 4D).

**FIGURE 5:**
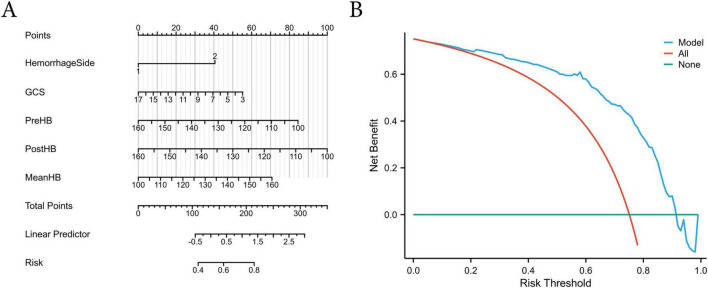
Nomogram and DCA of the final predictive model. **(A)** Nomogram integrating perioperative hemoglobin levels and clinical factors for individualized risk prediction; **(B)** DCA showing higher net clinical benefit of the composite model across a threshold probability range of 0.20–0.82.

To facilitate clinical application, a visual nomogram (Figure 5A) was constructed in this study. By combining patient clinical characteristics and HB indices, it enables the rapid assessment of individual poor functional outcome risk. Decision curve analysis (DCA) showed that, within a risk threshold of 0.20–0.82, the composite model had higher net benefit compared to the “all intervention” or “no intervention” strategies, indicating that the model effectively identifies high-risk patients and reduces unnecessary overtreatment, thereby improving the efficiency of interventions (Figure 5B).

## 4 Discussion

Basal ganglia hemorrhage represents a critical condition in neurosurgery. Approximately 70% of hypertensive intracerebral hematomas occur in the basal ganglia, and surviving patients often experience severe neurological sequelae (van Asch et al., 2010). To date, the surgical management of BGH remains controversial (Cordonnier et al., 2018; Seiffge et al., 2024). Notably, nearly one-third of patients with intracerebral hemorrhage experience hematoma expansion within 3–6 h after symptom onset, which is strongly associated with poorer clinical outcomes (Qureshi et al., 2001). Consequently, preventing hematoma growth has become a pivotal objective in the early management of intracerebral hemorrhage.

Our retrospective analysis of 213 patients demonstrated that perioperative HB indices–Pre-HB, Post-HB, and Mean-HB–were independently associated with postoperative prognosis in BGH. Patients with lower HB levels experienced delayed recovery, higher complication rates, and poorer long-term functional outcomes, as reflected by higher mRS scores. These findings are consistent with previous evidence linking anemia to adverse outcomes in cerebrovascular disorders and highlight the clinical significance of systematic HB monitoring. Importantly, this study is the first to investigate the prognostic value of dynamic perioperative hemoglobin fluctuations rather than relying on single-point measurements. Unlike prior studies that assessed only preoperative or postoperative HB levels (e.g., Moisa et al., 2025; Shah et al., 2023), our analysis incorporated multiple perioperative time points–Pre-HB, Post-HB, and Mean-HB–thereby capturing the physiological complexity of blood loss, transfusion response, and compensatory mechanisms. This dynamic evaluation offers a more comprehensive reflection of hemoglobin variability and its impact on neurological recovery, enhancing predictive precision and aligning with the paradigm of precision perioperative management. Building on these insights, we developed a composite predictive model that integrates HB indices with key clinical variables such as the GCS score. This integrated model demonstrated superior prognostic performance (AUC = 0.840) compared with single HB indices (AUC range: 0.599–0.623), providing a more accurate and clinically applicable tool for risk stratification in BGH patients. Our results align with prior evidence linking low HB with poor cerebrovascular outcomes. Wu et al. (2024) demonstrated that low Pre-Hb predicts postoperative infarction in moyamoya disease, while studies on intracerebral hemorrhage confirmed a linear relationship between decreased HB and adverse prognosis. These findings collectively underscore the clinical relevance of HB as a modifiable determinant influencing postoperative recovery, emphasizing that perioperative HB management may improve neurological outcomes (Acosta et al., 2021; Wu et al., 2024; Zhang et al., 2022).

The predictive model established in this study enables early stratification of patients at high risk for poor postoperative outcomes following BGH. Incorporating this nomogram into perioperative management may facilitate individualized therapeutic strategies. In particular, patients with lower HB-related risk scores or reduced GCS values should receive intensified perioperative optimization. From a hemoglobin management perspective, maintaining adequate oxygen-carrying capacity is vital for sustaining cerebral perfusion and minimizing secondary ischemic injury. For high-risk patients, the following measures should be considered: (1) early evaluation and correction of preoperative anemia through iron supplementation or erythropoietin administration when appropriate; (2) intraoperative monitoring of HB and hematocrit levels to guide timely transfusion or hemodilution correction; and (3) postoperative optimization of HB through minimization of blood loss, correction of coagulopathy, and implementation of individualized transfusion thresholds.

The integration of HB levels into the predictive model is particularly significant given the physiological role of HB in oxygen delivery and its interaction with multiple factors that affect recovery post-surgery. Low HB levels can exacerbate hypoxia (Gell, 2018), impair immune function (Bozza and Jeney, 2020; Buttari et al., 2013), and increase systemic inflammation (Gram et al., 2013; Tang et al., 2022), all of which may contribute to poor recovery outcomes in BGH patients. Therefore, early detection and intervention for anemia could enhance recovery by addressing these factors before they lead to severe complications.

Our model’s performance was further validated through calibration and decision curve analyses. Calibration curves showed a high degree of fit for the composite model, suggesting that it provides reliable predictions across various risk probabilities. The decision curve analysis further highlighted the practical utility of the model, demonstrating that it offers higher net benefits compared to conventional intervention strategies. By identifying high-risk patients more accurately, the model could help reduce unnecessary treatments and optimize resource allocation, leading to better outcomes with fewer complications.

Despite its promising results, this study has several limitations. First, the retrospective nature of the study may introduce bias, and the findings need to be validated through prospective trials. Additionally, the model’s application may be limited by the population it was derived from, as the cohort consisted of patients from a single institution. Thus, future studies should aim to validate the model across different institutions and populations to ensure its generalizability. Moreover, while HB levels are an important predictor, other biochemical and clinical variables, such as inflammatory markers and neuroimaging characteristics, should also be explored to further refine the model. Although previous observational studies have established an association between low Hb and poor cerebrovascular outcomes, the mechanistic pathways by which dynamic Hb changes influence recovery in basal ganglia hemorrhage remain unclear. Hence, the mechanistic significance of our findings should be interpreted with caution. Future experimental studies are needed to elucidate the biological processes underlying these associations.

In conclusion, this study provides compelling evidence for the inclusion of HB levels in the prognostic assessment of BGH patients. By combining HB data with other clinical indicators, we have developed a predictive model that can more accurately identify high-risk patients, enabling clinicians to tailor interventions and improve postoperative care. Future research should focus on validating this model in diverse populations and exploring additional factors that may enhance its predictive accuracy. The underlying mechanisms linking low Hb levels to unfavorable outcomes may involve Hb/heme-induced endothelial injury, disruption of the blood–brain barrier, and neuroinflammatory cascades. Experimental studies have shown that methemoglobin formation can trigger inflammation and immune-cell infiltration (Gram et al., 2013), while Hb/heme-mediated oxidative stress contributes to neuronal and vascular damage. Integrating these biological mechanisms enhances the plausibility of our findings and supports the potential of Hb modulation as a therapeutic strategy in BGH (Xia et al., 2022).

## Data Availability

The original contributions presented in this study are included in this article/supplementary material, further inquiries can be directed to the corresponding author.
